# Weight status and associated factors among HIV-infected people on antiretroviral therapy in rural Dikgale, Limpopo, South Africa

**DOI:** 10.4102/phcfm.v8i1.1230

**Published:** 2016-11-29

**Authors:** Felistas Mashinya, Marianne Alberts, Robert Colebunders, Jean-Pierre Van Geertruyden

**Affiliations:** 1 Department of Pathology and Medical Science, Faculty of Health Sciences, University of Limpopo, South Africa; 2 Global Health Institute, Faculty of Medicine, Antwerp University, Belguim

## Abstract

**Background:**

Underweight in human immunodeficiency virus (HIV)-infected people on antiretroviral therapy (ART) complicates the management of HIV infection and contributes to mortality, whereas overweight increases the risk of cardiovascular disease (CVD).

**Aim:**

The study determined weight status and associated factors in people with HIV infection receiving ART.

**Setting:**

Rural primary health care clinics in Dikgale, Limpopo province, South Africa.

**Methods:**

A cross-sectional study in which data were collected using the World Health Organization (WHO) stepwise approach to surveillance (STEPS) questionnaire and calculated using WHO analysis programmes guide. Weight and height were measured using standard WHO procedures, and body mass index was calculated as weight (kg)/height (m^2^). Data on ART duration were extracted from patients’ files. CD4 lymphocyte counts and viral load were determined using standard laboratory techniques.

**Results:**

Of the 214 participants, 8.9%, 54.7% and 36.4% were underweight, normal weight and overweight, respectively. Physical activity (OR: 0.99, *p* = 0.001) and male gender (OR: 0.29, *p* = 0.04) were negatively associated with overweight. Men who used tobacco were more likely to be underweight than non-tobacco users (OR: 10.87, *p* = 0.02). Neither ART duration nor viral load or CD4 count was independently associated with underweight or overweight in multivariate analysis.

**Conclusion:**

A high proportion of people on ART were overweight and a smaller proportion underweight. There is a need to simultaneously address the two extreme weight problems in this vulnerable population through educating them on benefits of avoiding tobacco, engaging in physical activity and raising awareness of CVD risk.

## Background

Overweight is a growing public health problem among HIV-infected people on ART, as also observed in the general populations worldwide.^1^ The increase in weight among HIV-infected people on ART has been attributed to a state of return to health in which appetite is gained and more food consumed, coupled with low physical activity.^1^ Weight gain in people on ART has been characterized by dorsocervical fat pad and visceral fat accumulation that may increase the risk of coronary artery disease.^2^ However, the relatively high percentage of people on ART that are underweight is still a concern.^3^ Being overweight or underweight may increase the risk of cardiovascular diseases.^4,5^

Although the developed world is faced with an increasing problem of overweight among HIV-infected people on ART,^6,7^ the developing world is experiencing a double burden of underweight and overweight.^8,9,10^ However, the prevalence of underweight and overweight reported from African countries is different,^8,9,10^ possibly due to differences in ART regimen, duration of ART and socio-economic factors.^2^

Reports on the burden of underweight and overweight among HIV-infected people from rural parts of South Africa are limited. Up-to-date information on the distribution of weight status among HIV-infected people can guide health providers in designing and improving health promotion programmes. Our goals were (i) to determine the prevalence of underweight and overweight in persons with HIV infection on ART in a rural area in the Limpopo province, one of the poorest provinces in South Africa, and (ii) to identify factors associated with being underweight and overweight in the study population.

## Methods

### Study design

The study was a descriptive cross-sectional study in which data were collected at one point in time. The study used quantitative methods that generated numerical data. Description of participants’ measurements and responses expressed as frequencies and categorised assisted in determining the factors associated with being underweight and overweight using regression models.

### Setting

The study was conducted in the three primary health care clinics, Seobi-Dikgale, Sebayeng and Dikgale that are situated within the Dikgale Health and Demographic Surveillance System (HDSS) site. The Dikgale HDSS site consists of 15 villages and is situated about 70 kms to the northeast of Polokwane, the capital city of Limpopo province. Limpopo province is one of the poorest provinces and is situated in the northern part of South Africa.

### Sample size calculation

The sample size was obtained using the formula for proportion in a cross-sectional study as $n=\frac{z^{2}p\left(1–p\right)}{d^{2}}$ where *n* = sample size, *Z* = a statistic for 95% confidence interval is 1.96, *P* = expected proportion, *d* = the maximum permissible difference between sample proportion and population proportion calculated as 20/100 x *P*.^11^

According to literature, the proportion of overweight in ARV-treated Africans ranges from 27.9% to 46.0%.^9,10^ Considering an average proportion of 36%, and a corresponding maximum margin of error of 7%, sample size was calculated as follows: Sample size $=\frac{(1.96{)}^{2}0.36\left(1–0.36\right)}{(0.07{)}^{2}}=181$ people

A 10% allowance was considered and the expected sample size was 200 people.

### Study population and sampling

The study population comprised of approximately 74 people with HIV infection treated at Seobi-Dikgale clinic, 373 people at Sebayeng and 377 people at Dikgale clinic. Based on these databases and using convenient sampling method, 20 people were recruited from Seobi-Dikgale, 96 people from Sebayeng and 98 from Dikgale clinics. A total of 214 participants were included in the study, and one was excluded due to pregnancy.

Participants aged 15 years and above who came to collect their ART were asked to participate in the study and were only included after written informed consent was obtained through the completion of a consent form approved by the MEDUNSA Ethics committee (-MREC/HS/137/2011:PG). In the case of minors, written informed consent was obtained from their legal guardians. Pregnant women were excluded.

### Data collection

Recruitment and clinical assessment were conducted from September 2013 to March 2014. The WHO stepwise approach to surveillance (STEPS) questionnaire^12^ was used to obtain information on socio-demographic characteristics, fruit and vegetable intake, physical activity, tobacco use and alcohol consumption. The questionnaire was first used on few people who did not form part of the study. Physical activity and fruit and vegetable intake levels were calculated using WHO analysis programmes guide.^13^ Physical inactivity was defined as < 600 metabolic equivalent task-minutes/week (MET-minutes/week), and low fruit and vegetable intake was defined as < 5 servings/day.^13^ Data on ART duration were extracted from patients’ files.

### Anthropometric measurements

Anthropometric measurements were taken following procedures as previously described.^14^ In brief, weight was measured to the nearest 0.1 kg, using Omron BF 400 (Omron Healthcare, Japan). Height was measured to the nearest 0.1 cm, using a stadiometer. Body mass index (BMI) was calculated as weight (kg)/height (m^2^). BMI is considered as underweight (< 18.5 kg/m^2^), normal (18.5–24.9 kg/m^2^), overweight (25.0–29.9 kg/m^2^) and obese (≥ 30.0 kg/m^2^).^15^ However, in this study, a BMI of 25.00 kg/m^2^ and above was considered overweight.

### Blood collection

Fasting venous blood samples were drawn by registered nurses. Whole blood was used to measure CD4 counts on the day of collection. Plasma from whole blood was separated through centrifugation at 2000 rpm for 15 minutes and stored at –80^°^C until viral load determination.

### Laboratory analysis

CD4 lymphocyte count was measured using the Pima Analyser (Inverness Medical, Tokyo, Japan). Viral load determination using the branched deoxyribonucleic acid (DNA) technique (Siemens, South Africa), was performed by Toga Molecular Biology and Pathology Medical Laboratory in South Africa.

### Statistical analysis

Statistical analysis was performed using Statistical Package for Social Sciences version 23.^16^ Variables were tested for normality using frequency histograms and line graphs. Data not normally distributed were logarithmically transformed for analysis. ANOVA was used to compare continuous variables and the chi-square test to compare categorical variables among underweight, normal weight and overweight groups. Bivariate logistic regression was used to determine individual influence of socio-demographic factors, HIV and ART variables on underweight and overweight status. Multivariate logistic regression modelling using a forward and backward stepwise approach was used to fit best predictive model and determine independent predictors. Factors significant at *p* ≤ 0.25 in univariate regression analysis and significant interaction terms at *p* < 0.05 were included in multivariate modelling.

### Ethical considerations

Ethical approval was obtained from the Ethics Committee of University of Limpopo, Medunsa campus (MREC/HS/137/2011: PG). Permission to conduct the study in the Dikgale HDSS clinics was obtained from the Department of Health-Provincial office and Primary Health Care Capricorn District office.

## Results

### Socio-demographic and lifestyle characteristics of participants

Of the 214 participants on ART, the majority were women (171 [79.9%]). The mean age of participants was 44.8 ± 11.8 years. Based on BMI, a few participants (19 [8.9%]) were underweight; 117 (54.7%) had a normal weight; and 78 (36.4%) were overweight. Underweight was three times more prevalent in men (18.6%) than in women (6.4%), whereas overweight was twice as prevalent in women (40.9%) than in men (18.6%). The prevalence of underweight among tobacco users was double the prevalence among non-tobacco users (15.6% vs. 7.1%), whereas the prevalence of overweight was higher among non-tobacco users compared with tobacco users (39.6% vs. 24.4%). However, the differences were not significant. A significantly higher proportion of physically inactive (< 600MET-minute/week) than physically active (< 600MET-minute/week) people were overweight (100% vs. 16%, *p* < 0.05). None of the people whose daily intake was more than 5 servings/day of fruits and vegetables were underweight (Table 1).

**TABLE 1:** Socio-demographic and lifestyle variables among HIV-infected people on ART by weight status.

Socio-demographic variable	All participants *N* = 214	Weight status			Linear association *p*
		Underweight *N* = 19	Normal weight *N* = 117	Overweight *N* = 78	
Age (years ± s.d.)	-	47.67 ± 14.22	44.23 ± 11.96	44.87 ± 10.60	0.51*
Age (years)					
< 30	11 (5.1)	1 (9.1)	6 (54.5)	4 (36.4)	-
30–39	69 (32.2)	4 (5.8)	43 (62.3)	22 (31.9)	0.88
40–49	64 (29.9)	6 (9.4)	31 (48.4)	27 (42.2)	-
≥ 50	70 (32.7)	8(11.4)	37 (52.9)	25 (35.7)	-
Gender					
Female	171 (79.9)	11 (6.4)	90 (52.6)	70 (40.9)	0.001
Male	43 (20.1)	8 (18.6)	27 (62.8)	8 (18.6)	-
Marital status					
Unmarried	164 (76.6)	15 (9.1)	90 (54.9)	59 (36.0)	0.75
Married	50 (23.4)	4 (8.0)	27 (54.0)	19 (38.0)	-
Education					
Primary only	114 (53.3)	11 (9.6)	61 (53.5)	42 (36.8)	0.92
Secondary and above	100 (46.7)	8 (8.0)	56 (56.0)	36 (36.0)	-
Employment					
Unemployed	166 (77.6)	17 (10.2)	87 (52.4	62 (37.3)	0.84
Employed	48 (22.4)	2 (4.2)	30 (62.5)	16 (33.3)	-
Tobacco use					
Yes	45 (21.0)	7 (15.6)	27 (60.0)	11 (24.4)	0.02
No	169 (79.0)	12 (7.1)	90 (53.3)	67 (39.6)	-
Alcohol use					
Yes	47 (22.0)	4 (8.5)	29 (61.7)	14 (29.8)	0.42
No	167 (78.0)	15 (9.0)	88 (52.7)	64 (38.3)	-
Pima Analyser (PA)					
< 600 MET-minutes/week	38 (24.2)	0 (0.0)	0 (0.0)	38 (100)	0.001
> 600 MET-minutes/week	119 (75.8)	14 (11.8)	86 (72.2)	19 (16.0)	-
Fruit and vegetable intake					
< 5 servings/day	150 (95.5)	14 (9.3)	83 (55.3)	53 (35.3)	0.19
> 5 servings/day	7 (4.5)	0 (0.0)	3 (42.9)	4 (57.1)	-

Data is presented as *n*(%) number (percentage) unless indicated.

* Analysis of variance test (ANOVA) *p*-value and chi-square test were used to obtain linear association *p*-value; s.d., standard deviation; ART, antiretroviral therapy; MET-minute/week, metabolic equivalent of task-minutes/week. BMI was considered as underweight (< 18.5 kg/m^2^), normal (18.5–24.9 kg/m^2^) and overweight (≥ 25.0 kg/m^2^). Data for Pima Analyser (PA) and fruit and vegetable are available for 157 participants.

### Clinical characteristics of participants

The majority of participants were on efavirenz-based ART (86%) and nevirapine-based ART (12.5%). Only three of the participants (1.5%) were on a lopinavir or ritonavir-based ART regimen. The mean duration of ART in this study was 36 months (range: 1–121 months). Majority of the participants (85%) had an undetectable viral load, and the mean CD4 count was 462 ± 235 cells/mm^3^.

The prevalence of underweight was lower in people with suppressed viral load (< 50 copies/ml) compared with people with a viral load of greater than 5000 copies/ml (7.3% vs. 20.0%, *p* > 0.05), whereas the prevalence of overweight was significantly higher in people with suppressed viral load (< 50 copies/ml) compared with people with a viral load of greater than 5000 copies/ml (39.9% vs. 0%, *p* < 0.05). Overweight people tended to have a higher mean CD4 count than the normal weight or underweight people, but levels of significance were not achieved. The levels of high sensitivity C-reactive protein were similarly high in underweight and in overweight people (7.01 mg/l vs. 5.75 mg/l, *p* > 0.05) but were significantly higher when compared with the levels in people with normal weight (Table 2).

**TABLE 2:** Clinical variables and conditions among HIV-infected people on ART by weight status.

Characteristics	All participants *N* = 214	Weight status			Linear association *p*
		Underweight *N* = 19	Normal weight *N* = 117	Overweight *N* = 78	
HIV and ART variables					
CD4 count (mean ± SD)	-	428.6 ± 255.6	432.7 ± 226.5	512.1 ± 237.1	0.06*
Cell/mm^3^	73 (35.4)	5 (6.8)	31 (42.5)	37 (50.7)	-
> 500	61 (29.6)	5 (8.2)	39 (63.9)	17 (27.9)	0.01
351–500	54 (26.2)	7 (13.0)	27 (50.0)	20 (37.0)	-
200–350	18 (8.7)	2 (11.1)	13 (72.2)	3 (16.7)	-
< 200					
Viral load ( copies/ml)					
< 50	178 (83.2)	13 (7.3)	94 (52.8)	71 (39.9)	-
51–500	15 (7.0)	3 (20.0)	9 (60.0)	3 (20.0)	0.008
501–5000	11 (5.1)	1 (9.1)	6 (54.5)	4 (36.4)	-
> 5000	10 (4.7)	2 (20.0)	8 (80.0)	0 (0.0)	-
ART duration (mean ± SD)		38.35 ± 25.71	33.76 ± 24.59	39.07 ± 23.77	0.30*
> 60 months	34 (17.3)	4 (11.8)	18 (52.9)	12 (35.3)	-
30–60 months	69 (35.2)	6 (8.7)	29 (42.0)	34 (49.3)	0.39
< 30 months	93 (47.4)	7 (7.5)	60 (64.5)	26 (28.0)	-
ART					
EFV-based ART	172 (86)	17 (9.9)	93 (54.1)	62 (36.0)	0.35
NVP-based ART	25 (12.5)	0(0.0)	15 (60.0)	10 (40.0)	-
Alluvia-based ART	3 (1.5)	1 (33.3)	0(0.0)	2 (66.7)	-
Clinical measurements and conditions					
Diabetes					
Yes	10 (4.7)	1 (10.0)	5 (50.0)	4 (40.0)	0.90
No	203 (95.3)	18 (8.9)	111 (54.7)	74 (36.5)	-
Hypertension					
Yes	56 (26.2)	3 (5.4)	27 (48.2)	26 (46.4)	0.06
No	158 (73.8)	16 (10.1)	90 (57.0)	52 (32.9)	-
HsCRP					
Normal level	71 (34.8)	3 (4.2)	52 (73.2)	16 (22.5)	0.046
High level	133 (65.2)	13 (9.8)	59 (44.4)	61 (45.9)	-
HsCRP: (mg/l)					
median(interquartile range)	-	7.01(3.22–15.6)	3.38(1.42–7.02)^b^	5.75(3.34–11.1)^b^	0.003*

* Analysis of variance test (ANOVA) *p*-value and chi-square test were used to obtain linear association *p*-value. ^b^, Indicates significantly different cells. Data are presented as *n*(%) - number (percentage) unless indicated. ART, antiretroviral therapy; BMI, body mass index; BMI was considered as underweight (< 18.5 kg/m^2^), normal (18.5–24.9 kg/m^2^) and overweight (≥ 25.0 kg/m^2^); HsCRP, high sensitivity C-reactive protein; NVP, nevirapine; EFV, efavirenz.

### Predictors of underweight in people on ART

In univariate analysis, gender (OR: 3.33, *p* = 0.02) and the interaction of gender and tobacco use (OR: 7.72, *p* = 0.001) were significantly associated with underweight. After controlling for employment, tobacco, fruit and vegetable intake, and viral load in multivariate model, the interaction of gender and tobacco use (OR: 10.87, *p* = 0.003) was an independent predictor of underweight in the final model (Table 3).

**TABLE 3:** Multivariate logistic regression analysis: predictors of underweight and overweight in HIV-infected people on ART.

Variables	Underweight				Overweight			
	Univariate		Multivariate		Univariate		Multivariate	
	Crude OR (95% CI)	*p*	AOR (95% CI)	*p*	Crude OR (95%CI)	*p*	AOR (95% CI)	*p*
Age (years)								
< 30	1 [Ref]	-	-	-	1 [Ref]	-	-	-
30–39	0.68 (0.06–6.08)	0.68	-	-	0.82 (0.22–3.09)	0.77	-	-
40–49	1.03(0.11–9.53)	0.98	-	-	1.28 (0.34–4.80)	0.72	-	-
≥ 50	1.29 (0.15–11.5)	0.82	-	-	0.97 (0.26–3.65)	0.97	-	-
Gender								
Female	1 [Ref]	*	-	-	1 [Ref]	*	1 [Ref]	-
Male	3.33 (1.25 - 8.87)	0.02	-	-	0.33 (0.14–0.75)	0.009	0.29(0.09–0.97)	0.04
Marital status								
Unmarried	1 [Ref]	-	-	-	1[Ref]	-	-	-
Married	0.86 (0.27–2.73)	0.80	-	-	1.09 (0.57–2.10)	0.95	-	-
Education								
Primary	1 [Ref]	-	-	-	1[Ref]	-	-	-
Secondary and above	0.81 (0.31–2.11)	0.67	-	-	0.96 (0.55–1.69)	0.90	-	-
Employment								
Unemployed	1 [Ref]	*	-	-	1 [Ref]	-	-	-
Employed	0.62 (0.29–1.31)	0.21	-	-	0.84 (0.43–1.65)	0.61	-	-
Tobacco use								
No	1 [Ref]	*	-	-	1 [Ref]	-	-	-
Yes	2.41 (0.89 – 6.53)	0.08	-	-	0.49 (0.23–1.04)	0.06*	-	-
Alcohol use								
No	1 [Ref]	-	-	-	1[ Ref]	-	-	-
Yes	0.94 (0.30–2.99)	0.92	-	-	0.68 (0.34–1.37)	0.28	-	-
Physical activity	(1.00–1.00)	0.26	-	-	0.99 (0.98–1.00)	0.001*	0.99 (0.99–1.00)	0.001
Fruit and vegetable intake	0.69 (0.37–1.29)	0.25*	-	-	1.11 (0.89–1.39)	0.37	-	-
CD 4 count (cell/mm^3^)								
> 500	1 [Ref]	-	-	-	1 [Ref]	*	-	-
351–500	1.21 (0.34–4.40)	0.77	-	-	0.38 (0.18–0.78)	0.008	-	-
201–350	2.02 (0.61–6.77)	0.25	-	-	0.57 (0.28–1.17)	0.13	-	-
< 200	1.70 (0.30–9.57)	0.55	-	-	0.20 (0.05–0.73)	0.02	-	-
Viral load (copies/ml)								
< 50	1 [Ref]	*	-	-	1 [Ref]	*	-	-
51–500	3.17 (0.79–12.68)	0.10	-	-	0.38 (0.10–1.38)	0.14	-	-
501–5000	1.27 (0.15–10.70)	0.83	-	-	0.86 (0.24–3.05)	0.82	-	-
> 5000	3.17 (0.61–16.51)	0.17	-	-		-	-	-
ART duration (months)								
> 60	1 [Ref]	-	-	-	1 [Ref]	*	-	-
30–60	0.71 (0.19–2.72)	0.62	-	-	1.78 (0.76–4.15)	0.18	-	-
< 30	0.61 (0.17–2.23)	0.46	-	-	0.71 (0.31–1.64)	0.43	-	-
Gender by tobacco use	7.72 (2.46–24.21)	0.001*	10.87(2.30–51.4)	0.003	-	-	-	-
*Classification accuracy*	-	-	*93.3*%	-	-	-	*82.7*%	-

OR, odds ratio; AOR, adjusted odds ratio; ART, antiretroviral therapy; CI, confidence interval;

* Variables that were included in the multivariate forward and backward stepwise regression model. Variables in final multivariate model are presented with adjusted odds ratio. BMI was considered as underweight (< 18.5 kg/m^2^), normal (18.5–24.9 kg/m^2^) and overweight (≥ 25.0 kg/m^2^).

### Predictors of overweight in people on ART

In univariate analysis, male gender (OR: 0.33, *p* = 0.009) and physical activity (OR: 0.99, *p* = 0.001) significantly associated negatively with overweight. After controlling for tobacco use, CD4 count, viral load and ART duration in multivariate model, male gender (OR: 0.29, *p* = 0.04) and physical activity (OR: 0.99, *p* = 0.001) were independent negative predictors of overweight. Neither ART duration nor viral load or CD4 count was independently associated with overweight in multivariate analysis (Table 3).

## Discussion

In this study, 8.9% of the participants were underweight and 36.4% were overweight. Similar to our finding, the prevalence of underweight in the general South African population was recently found to be 7.0%.^17^ However, the 50.8% prevalence of overweight in the general population^17^ was higher than the prevalence of overweight observed in our study. The prevalence of underweight and overweight among HIV-infected people on ART reported from rural KwaZulu Natal in South Africa^10^ are similar to the prevalence observed in our study. However, some low- to middle-income countries are still faced with a higher burden of underweight than overweight (Table 4). Although there is a greater tendency to gain weight while taking ART, weight loss has been observed,^3^ and therefore, health professionals should monitor patients’ weight. An increase in weight among people on ART may be due to a return to good health in which the appetite and caloric intake increases accompanied with a lack of physical activity.^1^ On the contrary, literature suggests that weight loss during the first 3 months after initiation of ART was associated with symptoms such as difficulty in breathing, nausea, vomiting and oral opportunistic infections.^3^ Oral opportunistic infections can limit the ability to chew and swallow food leading to loss of appetite and reduced caloric intake. Additionally, poor adherence to ART or drug failure that may result in disease progression may explain a weight loss in people on ART.^3^ However, our study did not evaluate these symptoms or drug adherence. In the United States^6^ and the United Kingdom,^7^ underweight was observed in ≤ 1% and overweight in ≥ 50% of people on ART (Table 4). Although an increasing number of people on ART in developed countries are overweight, in developing countries the co-existence of underweight and overweight among HIV-infected people on ART is a burden that is far from over, despite efforts to eradicate malnutrition. Fears are that the problem of malnutrition (underweight) may be compounded in the future by climate change impacting on agricultural yields and food security.^23^ Underweight among patients on ART in low- to middle-income countries was shown to contribute to excess early mortality, possibly due to poor immune reconstitution arising from deficient nutritional status.^24^ Similar to the general population,^4,5^ overweight in people on ART may increase the risk of developing CVD. The levels of C-reactive protein an inflammatory marker were similar in the underweight group and in the overweight group, as these extreme weight statuses are characterized by inflammation.^25^ Adipose tissue in overweight individuals may produce proinflammatory cytokine such as interleukin-6, a significant stimulator of C-reactive protein,^26^ whereas opportunistic infections that may be present in underweight individuals are associated with increased levels of C-reactive protein.^27^ However, individuals with very low CD4 counts less than 50 cells/µl have been observed to produce a small C-reactive response.^27^

**TABLE 4:** Prevalence of underweight and overweight in patients on ART in previous studies.

Author	Study design/Country	Characteristics of participants	Underweight (%)	Overweight (%)
Crum-Ciaflone et al., 2008^6^	Retrospective/USA	- Men	1	63
		- 18 years and above		
		- Mean ART duration 54 months		
McCormick et al., 2014^7^	A whole year survey/United Kingdom	- Men and women	African Black: 0.4	African Black: 77
		-18 years and above	F-1; M-0	F- 80.6; M- 70
		-Any duration of ART	White Caucasian- 1.7	White Caucasian- 49.2
			F- 4; M-1	F- 52; M- 48.7
Wasie et al., 2010^8^	Cross-sectional/northw est Ethiopia	- Men and women	27.8	4.8
		- 15 years and above		
		-Any duration of ART		
Mustapha et al., 2011^9^	Cross- sectional/Nigeria	- Males and females	24.1	27.9
		- 18 to 50 years old		
		- ART duration ≥ 6 months		
Malaza et al., 2012^10^	Population survey/South Africa	- Men and women	7.1	46
		- Adults	F-5.5; M-14.4	F-53.1; M-15.5
		- Any duration of ART		
de Senna et al., 2014^18^	Cross-sectional/Brazil	- Men and women	5.5	37.1
		- 20 years and above	F-7.6; M-3.8	F-42.9; M-32.3
Frantz & Murenzi, 2013^19^	Cross-sectional/Rwand a	- Males and females	F- 4; M- 1.1	F- 46; M-22.1
		- 18 years and above		
		- ART duration ≥ 12 months		
Gedle et al., 2015^20^	Cross- sectional/southern Ethiopia	- Men and women	25.2	NA
		- 18 years and above	F-24.9; M-25.9	
		- Any duration of ART		
Anand & Puri, 2014^21^	Cross- sectional/India	- Men and women	40.0	15.0
		- 21 years and above		
		- ART duration ≤ 6 months		
Mehta & Khatri, 2013^22^	Cross- sectional/Nepal	- Men and women	24.7	10.6
		- 19–73 years old		
		- ART duration ≥ 3 months		

F, female; M, male; ART, antiretroviral therapy; NA, not assessed. Underweight (< 18.5 kg/m^2^), and overweight (≥ 25.0 kg/m^2^).

In this study, overweight was disproportionately more frequent in women than in men, whereas underweight was more frequent in men than in women, as was reported among HIV-infected people in KwaZulu Natal, South Africa^10^ and in the general population of South Africa.^17^ In contrast, studies from Brazil^18^ and Rwanda^19^ reported a higher prevalence of underweight and overweight in women than men. In Ethiopia, the prevalence of underweight was similar among women and men.^20^

Physical inactivity^28^ and low tobacco use^29,30^ may contribute to the higher prevalence of overweight in women on ART. In addition, studies among black African women from sub-Saharan Africa suggest high target ‘ideal’ weight, less dissatisfaction with larger body shape and poor awareness of CVD risk as main contributing factors to overweight among HIV-infected women on ART.^7,17,31^ Black African men on ART on the contrary are less keen to be obese.^7^

Our study showed that men who used tobacco were more likely to be underweight than men who did not use tobacco. Further analysis to explain this increased risk of underweight among smokers showed that a higher proportion of smokers than non-smokers (11.8% vs. 7.7%) had a viral load greater than 5000 copies/ml and were physically active (100% vs. 95%). Previously, HIV infection was shown to increase the risk of underweight through increased resting energy expenditure,^3^ whereas the use of tobacco was shown to reduce appetite.^32^ People on ART who were not physically active were more likely to be overweight than those physically active as was also observed in Rwanda.^19^ Physical activity may reduce the risk of being overweight^33^ and has a potential anti-inflammatory effect in people on ART.^34^

In our study, neither ART duration nor viral load or CD4 count was independently associated with underweight or overweight. Similarly, a study from United States showed that demographics and ART duration could not independently predict the increase of weight in people on ART.^6^ The relationship between ART duration and BMI is inconsistent.^6,35,36^ In a longitudinal study, weight gain was observed in a short ART duration (24 weeks) that was followed by weight loss in people treated with stavudine-based ART and maintained weight gain in people treated with tenofovir after 144 weeks.^37^ Therefore, according to Crum-Cianflone et al. (2008),^6^ the differences in ART duration may account for variations in this relationship between ART duration and BMI.

According to the WHO strategy to tackle nutritional problems, stakeholders at global, regional and local levels have a mandate to improve diets and physical activity patterns of the population.^38^ In line with this strategy, South Africa introduced the ‘Move for your Health’ programme aimed at promoting physical activity. An initiative of major private health insurers the ‘Wellness programme’ rewards and recognizes those maintaining a healthy weight.^39^ In addition, programmes such as social grants, the provision of nutritional supplements to children and those on ART are aimed at alleviating undernutrition. However, the impact and effectiveness of these initiatives remains unclear.

Our study is one of a few in South Africa to provide information on the problem of underweight among people on ART in a rural area. The limitations of this study include its cross-sectional design that limits inference. In addition, fruit and vegetable intake, physical activity, tobacco and alcohol use were self-reported, and recall bias may have influenced results. Although our study did not assess the full dietary intake, an assessment of fruit and vegetable intake showed that people in all weight status categories had a lower fruit and vegetable daily intake than recommended. In agreement to this finding, a study conducted in a South African rural and semi-rural area, similar to Dikgale HDSS, found that the intake of fruit and vegetables in that community was low.^40^ Overcoming this problem particularly in rural areas may therefore remain a challenge, as evidence suggests an association of perceived expense, and low income with low fruit and vegetable intake.^40,41^ A low level of fruit and vegetable intake may lead to deficiencies in vitamins, predisposing people to a wide range of conditions including CVD, cancers and cataracts^41,42^ and may accelerate HIV disease progression.^43^ Most of our participants were older people as the majority of young adults live away from home as temporary migrants for employment reasons.^44^ Convenient sampling was used to recruit participants. However, recruitment was conducted for a whole month cycle, giving all patients collecting their medication equal opportunity to participate. We also acknowledge the small sample size of our study. Further longitudinal cohort intervention studies that would monitor weight changes in people on ART are needed.

## Conclusion

In a rural community in the Limpopo province of South Africa, a high proportion of people on ART were overweight and smaller proportions were underweight. Men who used tobacco had a higher risk of being underweight than non-tobacco users. Female gender and physical inactivity increased the risk of being overweight. There is need to simultaneously address the two extreme weight problems in people on ART through educating this vulnerable population on the benefits of staying away from tobacco use, engaging in physical activity and increasing awareness on CVD risk.

## References

[1] Crum-Ciaflone N, Roediger MP, Eberly L, et al Increasing rates of obesity among HIV-infected persons during the HIV epidermic. PLoS One. 2010;5(4):1–9. 10.1371/journal.pone.0010106 PMC2856157 20419086

[2] Cofrancesco J Jr., Freedland E, McComsey G Treatment options for HIV-associated central fat accumulation. AIDS Patient Care STDS. 2009;23(1):5–18. http://dx.doi.org/10.1089/apc.2008.0067 19055407 10.1089/apc.2008.0067 PMC10204865

[3] Li N, Spiegelman D, Drain P, et al Predictors of weight loss after HAART initiation among HIV-infected adults in Tanzania. AIDS. 2012;26(5):577–585. http://dx.doi.org/10.1097/QAD.0b013e32834f9851 22156968 10.1097/QAD.0b013e32834f9851

[4] Suastika K, Dwipayana P, Sarasati MR, et al Underweight is an important risk factor for coronary heart disease in the population of Ceningan Island, Bali. Diab Vasc Dis Res. 2012;9(1):75–77. http://dx.doi.org/10.1177/1479164111422828 21994166 10.1177/1479164111422828

[5] Tchernof A, Després JP Pathophysiology of human visceral obesity: An update. Physiol Rev. 2013;93:359–404. http://dx.doi.org/10.1152/physrev.00033.2011 23303913 10.1152/physrev.00033.2011

[6] Crum-Cianflone N, Tejidor R, Medina S, Barahona I, Ganesan A Obesity among HIV patients: The latest epidemic. AIDS Patient Care STDS. 2008;22(12):925–930. http://dx.doi.org/10.1089/apc.2008.0082 19072098 10.1089/apc.2008.0082 PMC2707924

[7] McCormick CL, Francis AM, Iliffe K, et al Increasing obesity in treated female HIV patients from sub-Saharan Africa: Potential causes and possible targets for intervention. Front Immunol. 2014;5:1–6. http://dx.doi.org/10.3389/fimmu.2014.00507 25431572 10.3389/fimmu.2014.00507 PMC4230180

[8] Wasie B, Kebede Y, Yibrie A Nutritional status of adults living with HIV/AIDS at the university of Gondar referral hospital, Northwest Ethiopia. Eur J Biol Sci. 2010;3(1):1–11.

[9] Mustapha KB, Ehianeta TS, Kirim RA, Osungwu FT, Oladepo DK Highly active antiretroviral therapy (HAART) and body mass index (BMI) relationship in people living with HIV/AIDS (PLWHA) in the Federal Capital Territory, Nigeria and the neighbouring states. J AIDS HIV Res. 2011;3(3):57–62.

[10] Malaza A, Mossong J, Bärninghausen T, Newell M Hypertension and obesity in a high HIV prevalence rural area in South Africa. PloS One. 2012;7(10):1–6. http://dx.doi.org/10.1371/journal.pone.0047761 10.1371/journal.pone.0047761 PMC3474786 23082211

[11] Naing L, Winn T, Rusli BN Practical issues in calculating the sample size for prevalence studies. Arch Orofacial Sci. 2006;1:9–14.

[12] World Health Organization Chronic diseases and health promotion: STEPwise approach to surveillance (STEPS). [cited 2014 January 23] http://www.who.int/chp/steps/en/

[13] World Health Organization Analysis programs documentation. WHO Steps surveillance. [cited 2015 November 15] http://www.who.int/chp/steps/resources/Analysis_Programs_Documentation_v2.0.pdf

[14] Mashinya F, Alberts M, Colebunders R, Van Geertruyden JP Cardiovascular risk factors in a treatment-naïve, human immunodeficiency virus-infected rural population in Dikgale, South Africa. S Afr Fam Pract. 2014;56(3):1–6.

[15] World Health Organization Physical status: The use and interpretation of anthropometry. Geneva: WHO; 1995 [cited 2013 January 25] http://www.who.int/childgrowth/publications/physical_status/en/8594834

[16] International Business Machines (IBM) Corp IBM SPSS Statistics for Windows, version 23.0. Armonk, NY: IBM Corp; 2014.

[17] Mchiza ZJ, Parker W, Makoae M, Sewpaul R, Kupamupindi T, Labadarios D Body image and weight control in South Africans 15 years or older: SANHANES-1. BMC Public Health. 2015;15:992 http://dx.doi.org/10.1186/s12889-015-2324-y 26423378 10.1186/s12889-015-2324-y PMC4588465

[18] de Senna AFK, de Oliveira SA, Velarde LGC, Setubal S Nutritional status of HIV positive patients in Niteroi, Rio de Janeiro, Brazil. J Health Popul Nutr. 2014;32(4):595–599. 25895192 PMC4438689

[19] Frantz JM, Murenzi A The physical activity levels among people living with human immunodeficiency virus/acquired immunodeficiency syndrome receiving high active antiretroviral therapy in Rwanda. SAHARA J. 2013;10(3–4):113–118. http://dx.doi.org/10.1080/17290376.2014.886081 24521093 10.1080/17290376.2014.886081 PMC4039135

[20] Gedle D, Gelaw B, Muluye D, Mesele M Prevalence of malnutrition and its associated factors among adult people living with HIV/AIDS receiving anti-retroviral therapy at Butajira Hospital, southern Ethiopia. BMC Nutr. 2015;1:5 http://dx.doi.org/10.1186/2055-0928-1-5

[21] Anand D, Puri S Anthropometric and nutritional profile of people living with HIV and AIDS in India: An assessment. Indian J Community Med. 2014;39(3):161–168. http://dx.doi.org/10.4103/0970-0218.137153 25136157 10.4103/0970-0218.137153 PMC4134532

[22] Mehta RS, Khatri S Nutritional status of the people living with AIDS receiving anti-retroviral therapy in eastern Nepal. [cited 2015 July 15]. Available from: http://www.conference.bonfring.org/papers/rajagiri_dyuti2013/dyuti18.pdf

[23] Omoyo NN, Wakhungu J, Oteng’I S Effects of climate variability on maize yield in the arid and semi-arid lands of lower eastern Kenya. Agr Food Secur. 2015;4:8 http://dx.doi.org/10.1186/s40066-015-0028-2

[24] Sicotte M, Langlois EV, Aho J, Ziegler D, Zunzunegui MV Association between nutritional status and the immune response in HIV positive patients under HAART: Protocol for a systematic review. Syst Rev. 2014;3:9–17. http://dx.doi.org/10.1186/2046-4053-3-9 24513015 10.1186/2046-4053-3-9 PMC3922999

[25] Stavropoulos-Kalinoglou A, Metsios GS, Panoulas VF, et al Underweight and obese states both associate with worse disease activity and physical function in patients with established rheumatoid arthritis. Clin Rheumatol. 2009;28(4):439–444. http://dx.doi.org/10.1007/s10067-008-1073-z 19096748 10.1007/s10067-008-1073-z

[26] Aronson D, Bartha P, Zinder O, et al Obesity is the major determinant of elevated C-reactive protein in subjects with the metabolic syndrome. Int J Obes. 2004;28:674–679. http://dx.doi.org/10.1038/sj.ijo.0802609 10.1038/sj.ijo.0802609 14993913

[27] Drain Paul K, Kupka R, Msamanga GI, Urassa W, Mugusi F, Fawzi WW C-reactive protein independently predicts HIV-related outcomes among women and children in a resource-poor setting. AIDS. 2007;21(15):2067–2075. http://dx.doi.org/10.1097/QAD.0b013e32826fb6c7 17885297 10.1097/QAD.0b013e32826fb6c7 PMC4005838

[28] Kim HK, Kim MJ, Park CG, Kim HO Gender differences in physical activity and its determinants in rural adults in Korea. J Clin Nurs. 2010;19(5–6):876–883. http://dx.doi.org/10.1111/j.1365-2702.2009.03054.x 20500331 10.1111/j.1365-2702.2009.03054.x

[29] Batista JDL, Militão de Albuquerque Mde F, Ximenes RA, et al Prevalence and socioeconomic factors associated with smoking in people living with HIV by sex, in Recife, Brazil. Rev Bras Epidemiol. 2013;16(2):432–443. http://dx.doi.org/10.1590/S1415-790X2013000200018 24142014 10.1590/S1415-790X2013000200018

[30] Gilani SI, Leon DA Prevalence and sociodemographic determinants of tobacco use among adults in Pakistan: Findings of a nationwide survey conducted in 2012. Popul Health Metr. 2013;11:16–26. http://dx.doi.org/10.1186/1478-7954-11-16 24004968 10.1186/1478-7954-11-16 PMC3850735

[31] Hurley E, Coutsoudis A, Giddy J, Knight SE, Loots E, Esterhuizen TM Weight evolution and perceptions of adults living with HIV following initiation of antiretroviral therapy in a South African urban setting. S Afr Med J. 2011;101(9):645–650. 21920157

[32] Chiolero A, Faeh D, Paccaud F, Cornuz J Consequences of smoking for body weight, body distribution, and insulin resistance. Am J Clin Nutr. 2008;87:801–809. 18400700 10.1093/ajcn/87.4.801

[33] Segatto AFM, Junior IFF, dos Santos VR, et al Lipodystrophy in HIV/AIDS patients with different levels of physical activity while on antiretroviral therapy. Rev Soc Bras Med Trop. 2011;44(4):420–424. http://dx.doi.org/10.1590/S0037-86822011000400004 21860886 10.1590/s0037-86822011000400004

[34] d’Ettorre G, Ceccarelli G, Giustini N, Mastroianni CM, Silvestri G, Vullo V Taming HIV-related inflammation with physical activity: A matter of timing. AIDS Res Hum Retroviruses. 2014;30(10):936–944. http://dx.doi.org/10.1089/aid.2014.0069 25055246 10.1089/aid.2014.0069 PMC4179917

[35] Silva M, Skolnik PR, Gorbach SL, et al The effect of protease inhibitors on weight and body composition in HIV-infected patients. AIDS. 1998;12:1645–1651. http://dx.doi.org/10.1097/00002030-199813000-00012 9764784 10.1097/00002030-199813000-00012

[36] Akinboro AO, Onayemi O, Ayodele OE, Mejiuni AD, Atiba AS The impacts of first line highly active antiretroviral therapy on serum selenium, CD4 count and body mass index: A cross sectional and short prospective study. Pan Afr Med J. 2013;15:1–9. http://dx.doi.org/10.11604/pamj.2013.15.97.2524 24198891 10.11604/pamj.2013.15.97.2524 PMC3810121

[37] Gallant JE, Staszewski S, Pozniak AL, et al Efficacy and safety of tenofovir DF vs. stavudine in combination therapy in antiretroviral-naive patients: A 3-year randomized trial. JAMA. 2004;292(2):191–201. http://dx.doi.org/10.1001/jama.292.2.191 15249568 10.1001/jama.292.2.191

[38] World Health Organization Obesity and overweight. [cited 2015 November 19]. http://www.who.int/mediacentre/factsheets/fs311/en/

[39] Goedecke JH, Jennings CL, Lambert EV Obesity in South Africa in Chronic diseases of Lifestyle in South Africa since 1995–2005. 2007 [cited 2014 April 30] http://www.mrc.ac.za/chronic/cdlchapter7.pdf

[40] Peltzer K, Promtussananon S Knowledge, barriers, and benefits of fruit and vegetable consumption and lay conceptions of nutrition among rural and semi-urban Black South Africans. Psychol Rep. 2004;94(3):976–982. http://dx.doi.org/10.2466/pr0.94.3.976-982 15217058 10.2466/pr0.94.3.976-982

[41] Oguntibeju OO, Truter EJ, Esterhuyse AJ The role of fruit and vegetable consumption in human health and disease prevention. Diabetes Mellitus – Insights and Perspectives, Prof. Oluwafemi Oguntibeju, ISBN: 978-953-51-0939-6, InTech; 2013 http://dx.doi.org/10.5772/50109

[42] Peltzer K, Phaswana-Mafuya N Fruit and vegetable intake and associated factors in older adults in South Africa. Glob Health Action. 2012;5:18668 http://dx.doi.org/10.3402/gha.v5i0.18668 10.3402/gha.v5i0.18668 PMC3511777 23195518

[43] Nkengfack GN, Ngogang J, Englert H Effects of ‘5 a day’ fruit and vegetable intake on micronutrient level and oxidative stress markers in HIV positive patients: A cluster randomized trial. Oxid antioxid Med Sci. 2013;2(4):275–284. http://dx.doi.org/10.5455/oams.270713.or.052

[44] Alberts M, Dikotope SA, Choma SR, et al Health and demographic surveillance system profile: The Dikgale Health and Demographic Surveillance System. Int J Epidemiol. 2015;1–7. http://dx.doi.org/10.1093/ije/dyv157 26275454 10.1093/ije/dyv157

